# Impact of Acute Malaria on Pre-Existing Antibodies to Viral and Vaccine Antigens in Mice and Humans

**DOI:** 10.1371/journal.pone.0125090

**Published:** 2015-04-28

**Authors:** Simran Banga, Jill D. Coursen, Silvia Portugal, Tuan M. Tran, Lisa Hancox, Aissata Ongoiba, Boubacar Traore, Ogobara K. Doumbo, Chiung-Yu Huang, John T. Harty, Peter D. Crompton

**Affiliations:** 1 Department of Microbiology, University of Iowa, Iowa City, Iowa, United States of America; 2 Laboratory of Immunogenetics, National Institute of Allergy and Infectious Diseases, National Institutes of Health, Rockville, Maryland, United States of America; 3 Mali International Center of Excellence in Research, University of Sciences, Techniques and Technologies of Bamako, Bamako, Mali; 4 Division of Biostatistics and Bioinformatics, Sidney Kimmel Comprehensive Cancer Center, Johns Hopkins University, Baltimore, Maryland, United States of America; 5 Department of Pathology, University of Iowa, Iowa City, Iowa, United States of America; 6 Interdisciplinary Graduate Program in Immunology, University of Iowa, Iowa City, Iowa, United States of America; Centro de Pesquisa Rene Rachou/Fundação Oswaldo Cruz (Fiocruz-Minas), BRAZIL

## Abstract

Vaccine-induced immunity depends on long-lived plasma cells (LLPCs) that maintain antibody levels. A recent mouse study showed that *Plasmodium chaubaudi* infection reduced pre-existing influenza-specific antibodies—raising concerns that malaria may compromise pre-existing vaccine responses. We extended these findings to *P*. *yoelii* infection, observing decreases in antibodies to model antigens in inbred mice and to influenza in outbred mice, associated with LLPC depletion and increased susceptibility to influenza rechallenge. We investigated the implications of these findings in Malian children by measuring vaccine-specific IgG (tetanus, measles, hepatitis B) before and after the malaria-free 6-month dry season, 10 days after the first malaria episode of the malaria season, and after the subsequent dry season. On average, vaccine-specific IgG did not decrease following acute malaria. However, in some children malaria was associated with an accelerated decline in vaccine-specific IgG, underscoring the need to further investigate the impact of malaria on pre-existing vaccine-specific antibodies.

## Introduction

Since the Expanded Program on Immunization was implemented in the mid-1970s [1] there has been remarkable progress toward reducing the morbidity and mortality associated with vaccine-preventable diseases [2], yet ~20% of deaths in children under 5 years of age remain vaccine-preventable [3]. In lower-income regions, this is largely attributable to inadequate immunization coverage [4]; however, efforts to reduce vaccine-preventable deaths may be hindered further in malaria-endemic regions where it has long been suspected that *Plasmodium falciparum* infection reduces vaccine efficacy in children [5].

Most licensed vaccines confer protection through antibodies [6]. Long-lived antibody responses depend on memory B cells (MBCs) and long-lived plasma cells (LLPCs) [7]. LLPCs reside in the bone marrow and constitutively secrete antibodies, whereas MBCs mediate recall responses after antigen re-exposure by differentiating into antibody-secreting cells (ASCs). Although heterogeneity exists in the longevity of antibody responses following infection or vaccination, in general, antibody responses are long-lived. For example, half-life estimates of IgG responses in adults range from 11 years for the tetanus vaccine to 3014 years for the measles vaccine [8].

In contrast, antibody responses to *P*. *falciparum* infection are relatively short-lived, particularly in children [9], which likely contributes to the inefficient acquisition of clinical immunity to malaria [10]. Mounting evidence suggests that *Plasmodium* blood-stage infection is associated with dysregulated B cell and CD4^+^ helper T cell function and that this contributes to short-lived antibody responses to malaria [11–14]. What remains unknown is whether antibody levels induced by prior vaccinations are adversely affected by *P*. *falciparum* infections—a scenario that would dictate re-evaluation of policies regarding re-immunization frequency in malaria-endemic areas.

Interestingly, a recent study in mice suggested that *Plasmodium* blood-stage infection is deleterious to pre-existing levels of heterologous antibodies. Specifically, Ng *et al*. showed that *Plasmodium chaubaudi* (*Pcc*) blood-stage infection of influenza-immune B6 mice resulted in a transient drop in influenza-specific antibodies and ASCs in bone marrow that, paradoxically, recovered ~21 days post *Pcc* infection [15]. Given the potential public health implications of even transient declines in antibodies to common childhood vaccines, we sought to determine whether findings in the *Pcc* model are generalizable to other *Plasmodium* species in mice of diverse genetic backgrounds and, of clinical relevance, whether acute malaria in children is associated with a decrease in levels of pre-existing antibodies to vaccines.

## Materials and Methods

### Mice

BALB/c and C57BL/6 mice (NCI, Frederick, MD) and Swiss Webster mice (Harlan Laboratories) were housed at the University of Iowa. Female mice were used to initiate experiments at 8–12 weeks of age. Euthanasia was carried out by cervical dislocation. Mouse experiments were approved by The University of Iowa Animal Care and Use Committee.

#### Immunizations

SRBC immunization: 5 ml of Sheep blood (Colorado Serum Company, Denver, CO) was washed twice with Gibco PBS (Life Technologies). Red blood cells (RBCs) were pelleted at 1000 rpm for 10 min and the volume of the cell pellet measured. One volume of pellet was resuspended in 9 volumes PBS to prepare 10% SRBC suspension. 200 μl of the 10% SRBC suspension was injected intraperitoneally. Influenza virus immunization: Stock A/37/PR/8 (PR8) with TCID titers of 10^11^ /ml were diluted with PBS to 1:200,000 and 50 μl of viral suspension was given intranasally to anesthetized mice.

#### Infections and chloroquine treatment

Cryopreserved *P*. *yoelii* (*Py*) 17XNL infected RBCs were diluted with saline such that mice were intravenously injected with 100,000 *P*. *yoelii* (*Py*)-infected RBCs at the time indicated relative to immunization with SRBC or IAV. Parasitemia was monitored by Giemsa stained blood smears. In the indicated experiments, mice were treated on day 4 post infection and every second day thereafter by intraperitoneal injection with 50 mg/kg of chloroquine to prevent high parasite burden. Parasitemia in chlorquine-treated mice did not exceed 4% at any time point during the infection. *Listeria monocytogenes* (DPL-1942) was appropriately diluted and 5 x 10^5^ cfu were injected intravenously. LCMV Armstrong was appropriately diluted and 2 x 10^5^ pfu injected intraperitoneally. For challenge studies, PR8 stock virus was diluted 1:20,000 and mice were infected intranasally.

#### Hemagglutination antibody titer assay

Serum samples obtained at the indicated days relative to immunization or infection were serially diluted in PBS and mixed 1:1 with a 1% suspension of SRBC in triplicate in round-bottom 96-well plates. Plates were mixed and incubated at 4°C for 60 minutes then observed for hemagglutination. Hemagglutinin antibody titers are the highest dilution of serum with a positive result. Titers are expressed as logarithms to base 2 (log_2_).

#### Virus neutralizing antibody titers

Sera obtained at indicated days relative to immunization and infection were serially diluted in DMEM and 60 μl of each dilution (in triplicate) was mixed with equal volume containing 1000 TCID_50_ PR8 in a round-bottomed 96-well plate and incubated at 4°C for 20 minutes. 100 μl of this mixture was added to 96-well flat bottom plates containing 1 x 10^5^ MDCK cells per well. Plates were incubated for 24 hours in a tissue culture CO2 incubator, medium was replaced with 200 μl of DMEM containing 10% FCS, 0.002% Trypsin and incubated for 72 hours. To determine neutralizing titers, 100 μl of supernatant was mixed with equal volume of 1% chicken RBC suspension, incubated at 4°C for 30 minutes and observed for hemaagglutination as described above. The highest dilution of serum that neutralizes the virus and thus shows no agglutination is the neutralizing titer. Titers are expressed logarithms to base 2 (log_2_).

#### Virus burden in lungs

On day three post-challenge infection with PR8, lungs were homogenized in DMEM and stored at −80°C until use. Samples were thawed and virus levels determined as above.

#### Plasma cell Apoptosis, BAFF-R expression and BAFF level determination

Plasma cells in the spleen and bone marrow were identified with the indicated antibodies as CD45.2^+^ (104), B220^+^ (6B2), CD19^+^ (6D5), IgD^-^ (11-26c.2a), CD138^+^ (281–2). Apoptosis in plasma cells was determined by detection of active Caspase-3/7 using the VybrantFAMCaspase-3 and Caspase-7 Assay Kit (Invitrogen) according to manufacturer’s protocol. BAFF receptor (R) expression was detected on CD45.2^+^B220^+^CD19^+^IgD^-^CD138^+^ cells by staining with anti-BAFF-R mAb (11C1). Serum BAFF levels were quantified by ELISA according to manufacturer’s protocol (R&D Systems).

#### Ethical approval for Mali study

The Ethics Committee of the Faculty of Medicine, Pharmacy and Dentistry at the University of Sciences, Technique and Technology of Bamako, and the NIAID/NIH IRB approved this study. Written informed consent was obtained from parents or guardians of participating children.

### Mali study

The field study was conducted in Kalifabougou, Mali where *P*. *falciparum* transmission occurs annually (June-December). The cohort was described in detail elsewhere [10]. Here, we identified 54 children aged 2–5 years not infected with *P*. *falciparum* (by PCR) in May 2012 who had plasma available before (January 2012) and after (May 2012) the 6-month dry season, 10 days after the first malaria episode of the ensuing 6-month malaria season (variable date) and after the subsequent dry season (May 2013). Malaria was defined as ≥2,500 asexual parasites/μL, axillary temperature ≥37.5°C and no other cause of fever by exam. Malaria was detected by passive and weekly active surveillance. Individuals with malaria symptoms and any parasitemia were treated according to Malian National Malaria Control Program guidelines. Blood was drawn by venipuncture into sodium citrate–containing Vacutainer tubes (BD) and plasma separated and cryopreserved.

#### *P*. *falciparum* detection

Thick blood smears stained with Giemsa were counted against 300 leukocytes. Densities were recorded as the number of asexual parasites/microliter blood based on a mean leukocyte count of 7500 cells/μL. *P*. *falciparum* PCR methods were described previously [10].

#### ELISA human samples

IgG specific for tetanus toxoid, measles and Hepatitis B surface antigen was measured using ELISA kits according to manufacturer’s instructions (Alpha Diagnostic, San Antonio, TX). Plasma diluted 1:100 was analyzed in duplicate on single plates for each vaccine-specific ELISA. IgG concentrations were calculated by fitting to standard curves generated from standards in each kit and expressed as Units/milliliter.

### Statistical analysis

Mouse data were analyzed with two-tailed unpaired Student’s t-test using GraphPad Prism software. Humans ELISA data were log-transformed (with base 10). The Wilcoxon rank-sum test was used to compare slopes. Linear mixed-effects models compared rates of change during periods of malaria exposure and non-exposure, where the random intercept accounted for correlations among data points (before and after dry season, and 10 days after treatment) and fixed effects included time from enrollment and the minimum of 0 and time since end of dry season. Half-lives of IgG levels were estimated by fitting a linear mixed-effects model with three data points: before and after first dry season, and after second dry season, where random intercept accounted for within-subject correlation, and age at visit as fixed effect. IgG half-lives were estimated by the ratio of log_10_ (1/2) and fixed-effect slope of age; 95% CIs were obtained by similar calculations using endpoints of the 95% CI of fixed-effect slopes. Statistical significance was defined as 2-tailed *P* value<0.05. Analyses were performed in R v.2.15.1 (http://www.R-project.org) or Prism 5.0d (GraphPad).

## Results

### *Plasmodium* infection compromises antibody responses to third party antigens

**Fig 1:**
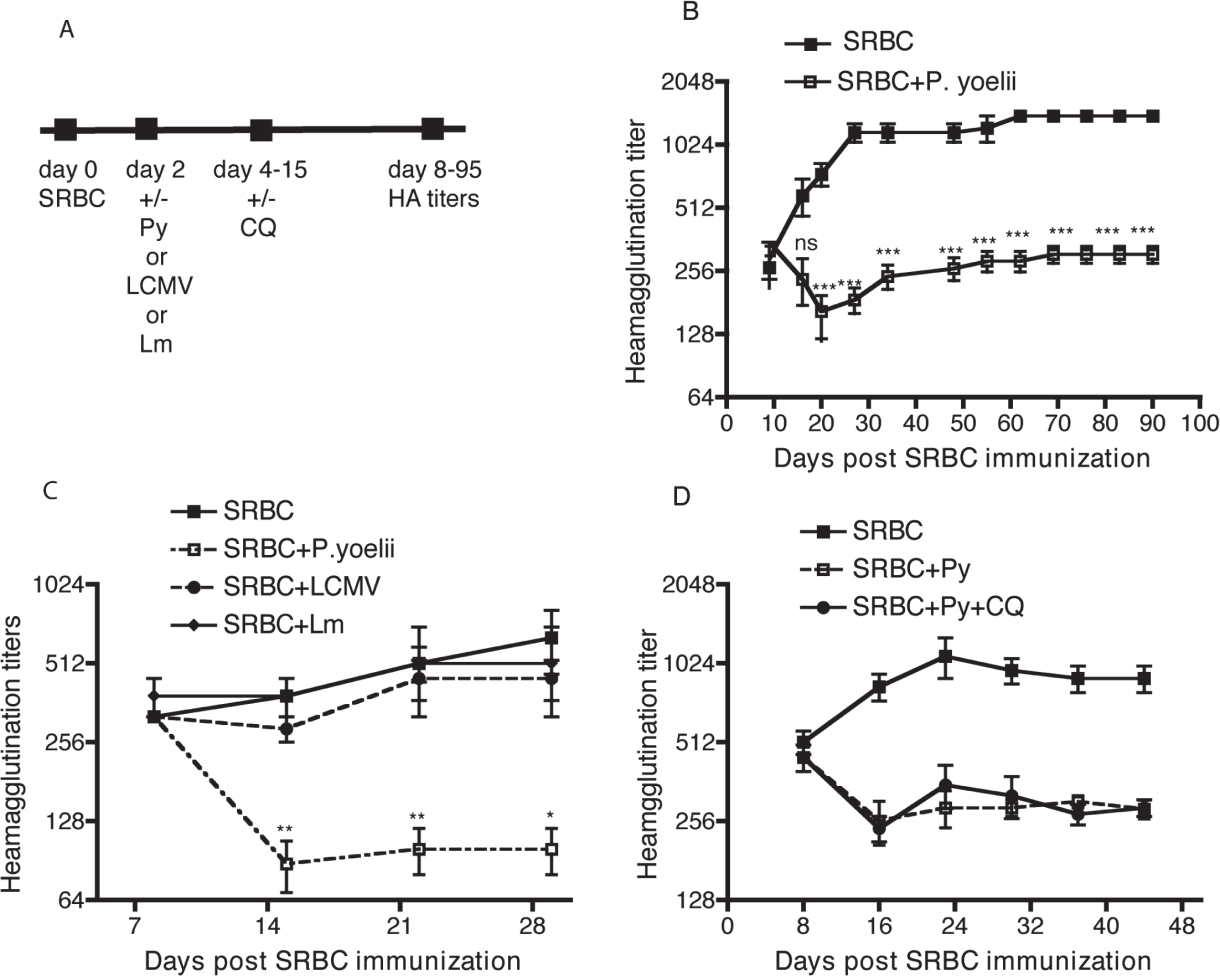
Decrease of SRBC-specific antibody responses after *P*. *yoelii* infection. **A**: Experimental design. Female BALB/c mice were immunized with 10% SRBC suspension intraperitoneally. Two days later, half of these mice were infected with 10^5^ *P*. *yoelii* infected RBCs intravenously, while the other half of mice were injected with saline. Serum samples were collected on specific days and analyzed for Heamagglutination titers. **B**: Heamagglutination titers over a period of 90 days in mice immunized with SRBC and infected or not infected with *P*.*yoelii*. **C**: SRBC immunized mice were injected with saline or infected after two days with *P*. *yoelii*, *L*. *monocytogenes* (5 X 10^6^ cfu) or LCMV (2 X 10^5^ pfu) and Heamagglutination titers were analyzed over a period of 30 days. **D**: SRBC immunized mice were injected with saline or infected after two days with *P*. *yoelii*. Some *P*. *yoelii* infected mice received chloroquine (50 mg per kg) on days 4, 7, 10, 12 and 15 post infection to limit blood stage parasitemia and Heamagglutination titers were analyzed over a period of 30 days. Parasitemia did not exceed 4% in the chloroquine treated mice. The data are representative of two or more independent experiments with10 mice per group/exp. *p<0.05, **p<0.01, ***p<0.001.

Multiple studies report atypical MBCs and relatively poor antibody responses in children living in malaria endemic regions [11]. However, it remains unknown if these aberrant responses are dictated at early or late time points during chronic *Plasmodium* infection and whether they reflect malaria-specific or generalized immune system abnormalities. To address this, we immunized BALB/c mice with Sheep Red Blood Cells (SRBC), then infected some mice in each group two days later with 10^5^ *P*. *yoelii* (Py) 17XNL parasitized red blood cells (pRBC) (Fig 1A). This experiment was designed to mimic natural infection, which is restricted to asymptomatic liver stage for ~44 hours in Py sporozoite-infected mice and is followed by blood-stage infection. SRBC-specific IgG titers were then determined at ~weekly intervals. This experiment revealed that anti-SRBC titers in control and Py-infected mice were similar at day 8 post-immunization (6 days post-infection), however, while titers continued to rise in control mice and stabilized by ~30 days post-immunization, anti-SRBC titers in Py-infected mice decreased over ~2 weeks before stabilizing for the 90-day evaluation period (Fig 1B). Importantly, replacing Py with bacterial (*Listeria monocytogenes*) or viral (lymphocytic choriomeningitis virus) infections did not compromise anti-SRBC antibody responses (Fig 1C). Thus, the failure to complete the normal antibody response to SRBC was not a general feature of infection. Additionally, treatment of mice with sub-clearing doses of the anti-malarial drug chloroquine (beginning 4 days post-infection and every 3 days following initial inoculation) limited infection (blood-stage parasitemia remained <4% for the entire experiment, data not shown) but did not prevent the Py associated drop in antibody titers (Fig 1D). Of note, similar data were generated in inbred C57BL/6 (B6) and outbred Swiss Webster mice (not shown). Thus, Py blood-stage infection rapidly and specifically impairs the evolution of antibody responses to a third-party antigen, in both inbred and outbred mice, and this does not require sustained high-level parasitemia.

### *Plasmodium* infection decreases pre-existing antibody titers and compromises immunity

**Fig 2:**
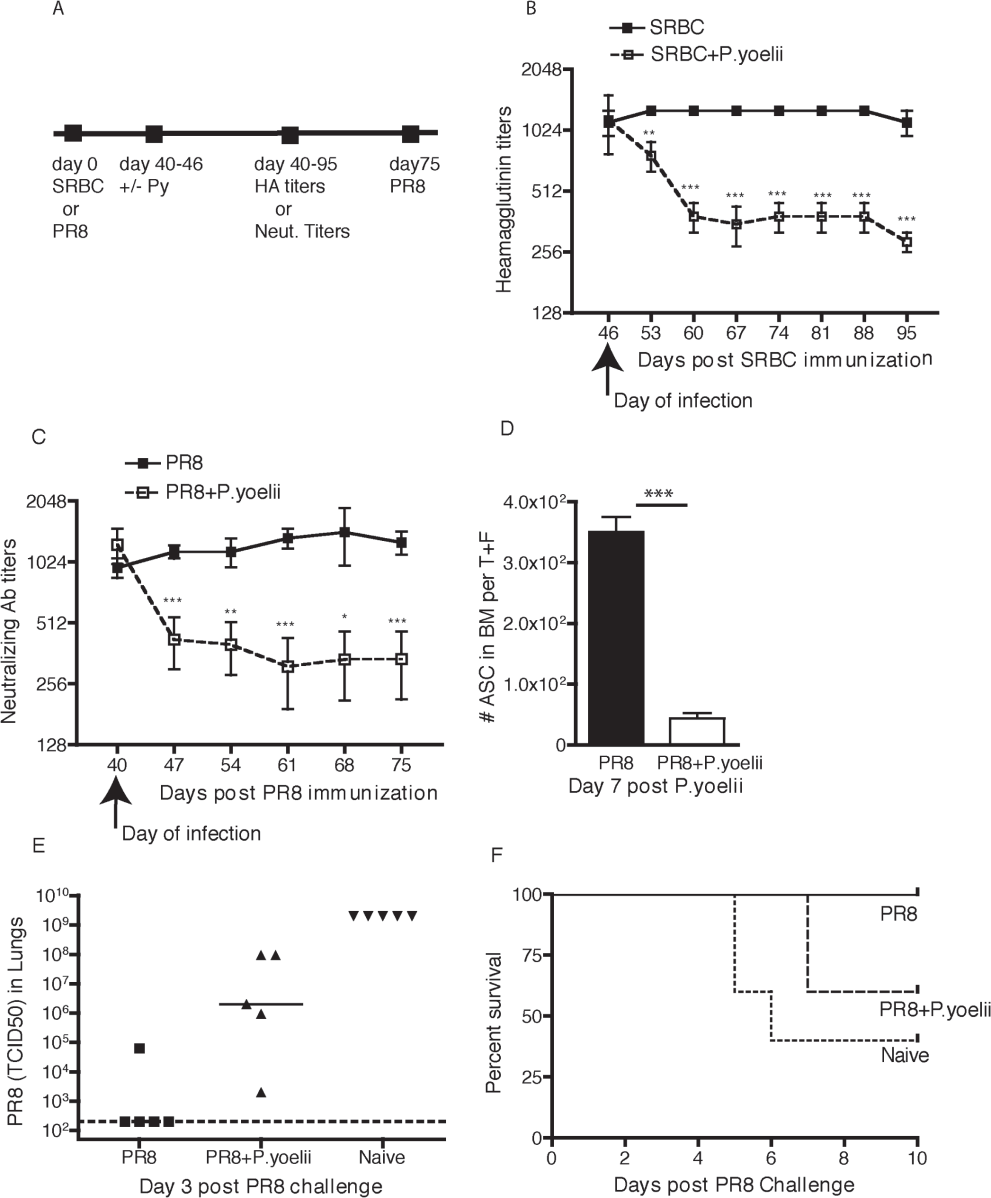
Decrease of preexisting antibodies after *P*. *yoelii* infection. **A**: Experimental design. BALB/c mice and outbred Swiss Webster mice were immunized with SRBC or received sublethal intranasal infection with PR8 IAV. At the indicated days post infection, some mice were infected with *P*. *yoelii*. Serum samples collected on specific days were analyzed for Heamagglutination titers or virus neutralizing antibody titers. **B**: Heamagglutination titers over a period of 95 days post SRBC immunization in *P*. *yoelii* infected and uninfected mice. **C**: Virus neutralizing antibody titers over a period of 75 days post PR8 infection in mice and infected or not infected with *P*.*yoelii*. **D**: PR8 specific Antibody secreting cells in the bone marrow of immune mice seven days after saline or *P*. *yoelii* infection. The numbers are derived from combination of one tibia and one femur from each mousse. **E**: Naïve or PR8 immune mice receiving saline or *P*. *yoelii* were challenged with PR8 virus 40 days post *P*.*yoelii* infection. On day 3 post challenge, lungs were harvested to determine virus load., each symbol represents an individual mouse. **F**: Survival curve of mice as in E. Data are representative of three independent experiments with 8 to 12 mice per group/experiment. * p<0.05, ** p<0.01, *** p<0.001.

A recent study showed that *Pcc* blood-stage infection of influenza virus-immune B6 mice resulted in a transient drop in circulating antibody levels and ASCs in bone marrow that, paradoxically, recovered by ~21 days post-infection [15]. To determine if this finding is generalizable to Py infection in a genetically diverse host, outbred Swiss Webster mice were immunized with SRBC and SRBC-specific antibody titers were determined until stable titers were observed (>40 days). On day 46 post-SRBC, some mice were infected with Py pRBC. Again, antibody titers fell sharply over the ensuing 2 weeks before stabilizing in Py-infected mice, whereas no drop in antibody titers was observed in control mice (Fig 2B). These data show that blood-stage Py infection also compromises stable antibody responses maintained by LLPCs. To extend these data to antibody responses against a pathogen and to compare with the published data on *Pcc* in B6 mice, outbred Swiss Webster mice were sublethally infected with the mouse adapted H1N1 influenza A virus (IAV) A/PR/8 (PR8). At day 40 post-infection, when PR8-specific neutralizing antibody titers had been stable for several weeks, some mice were infected with Py pRBC (Fig 2A). Strikingly, we observed a substantial decline in circulating IAV-specific neutralizing antibodies (Fig 2C) over the next two weeks, followed by stable maintenance at reduced amounts for at least 35 days post Py infection. Of note, analyses of IAV-specific ASCs in the bone marrow 7 days after infection revealed a significant decrease in mice that had received Py blood-stage infection (Fig 2D). To determine the biological import of decreased IAV-neutralizing antibodies, naïve, IAV-immune and IAV-immune/Py-infected mice were challenged with PR8 and virus titers in the lung (Fig 2E) and mortality (Fig 2F) were evaluated. As expected, naïve mice had high virus titers and ~50% mortality whereas control IAV-immune mice had virus titers in the lungs below the limit of detection and exhibited no mortality. In contrast, IAV-immune mice with diminished antibody responses due to Py infection exhibited substantial virus titers in the lung and ~40% mortality after homologous IAV challenge. These data reveal that the Py associated drop in long-term antibody titers has a profound impact on the ability of an immune host to resist subsequent infections.

**Fig 3:**
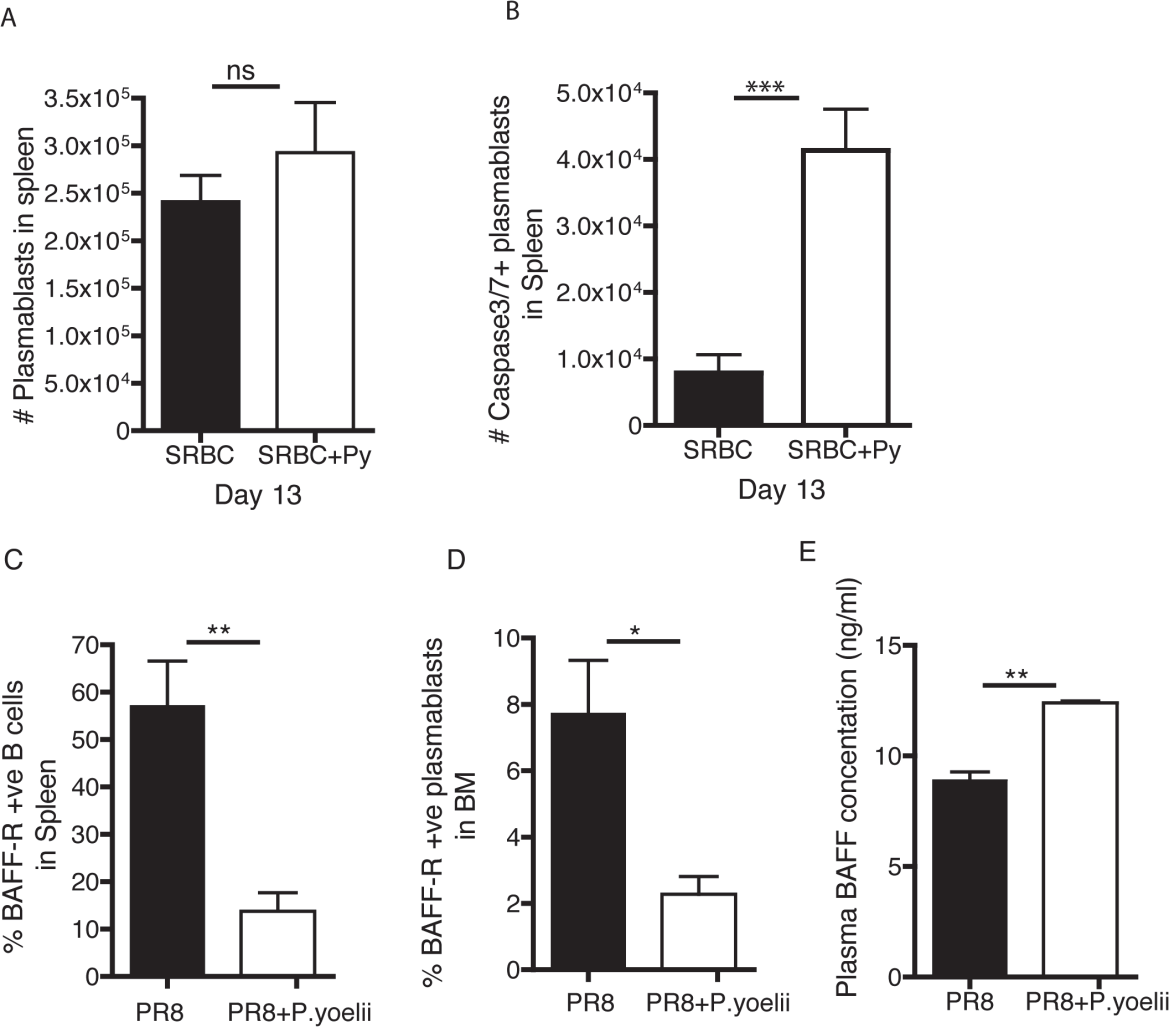
*P*. *yoelii* infection increases plasma cell apoptosis, decreases plasma cell BAFF-R and increases circulating BAFF concentrations. BALB/c mice were immunized with 10% SRBC suspension. Two days later, half of these mice were infected with 10^6^ *P*. *yoelii* parasitized RBC while the other half of mice were injected with saline. (A) Total plasma cells in the spleen at day 13 post SRBC immunization. (B) Total caspase 3/7 positive plasma cells in the spleen at day 13 post SRBC immunization. Data in A,B are mean + S.D. and represent one of two independent experiments with 5 mice per group. Swiss Webster mice were infected with a sublethal dose of PR8. Forty-two days later, half of these mice were infected with 10^6^ *P*. *yoelii* parasitized RBC. The percent of BAFF-R positive plasma cells in the (C) spleen and (D) bone marrow were determined on day forty-eight after immunization (day 6 after Py infection). Serum was obtained from mice in C,D and analyzed for circulating BAFF concentrations (E). Data in C-E reflect mean + S.D. and represent one of two independent experiments with 5 mice per group. *** p<0.001, ** p<0.01, * p<0.05, ns p non-significant

Of note, evaluation of splenic CD138^+^ plasma cells in SRBC-immunized mice with and without blood-stage Py infection revealed no changes in total cell numbers in the spleen, however, there was a substantial increase in the total numbers of plasma cells that exhibited signatures of apoptosis (active caspase 3/7) (Fig 3A and 3B). BAFF-receptor (BAFF-R) signals promote survival of antibody secreting plasma cells [16] and BAFF levels have been shown to be elevated in children with malaria (28). Influenza immune mice (day 42 p.i.) were mock infected or infected with Py and BAFF-R expression on plasma cells and circulating BAFF levels were analyzed six days later. We observed decreased numbers of BAFF-R expressing CD138^+^ plasma cells in spleen and bone marrow (Fig 3C and 3D) of Py infected mice. In addition, we observed increased amounts of BAFF in the serum (Fig 3E). The loss of BAFF-R may contribute to decreased survival of plasma cells observed with Py infection and explain the elevated BAFF levels in the circulation.

**Fig 4:**
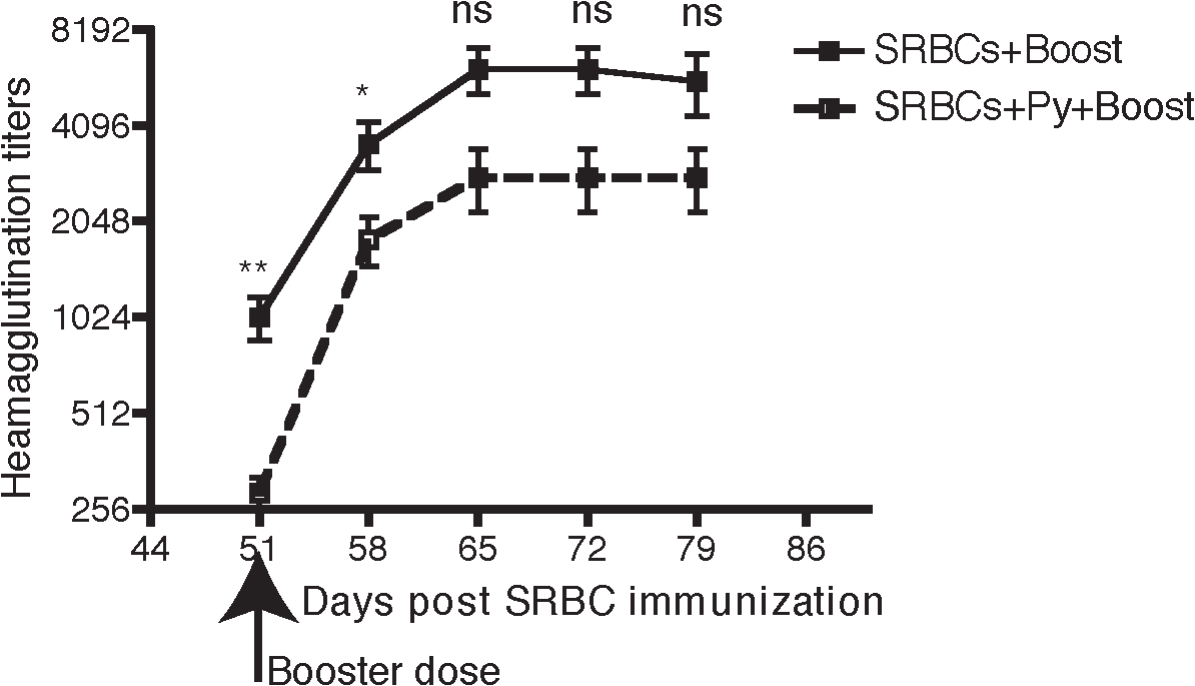
*P*. *yoelli* infection does not disrupt the anamnestic response to SRBC immunization. BALB/c mice were immunized with 10% SRBC suspension. Two days later, half of these mice were infected with 10^6^ *P*. *yoelii* while the other half of mice were injected with saline. A booster dose of 10% SRBC suspension was given after 40+ days to both groups of mice. Serum samples were collected on the indicated days and analyzed for Heamagglutinin antibody titers. Data are representative of two experiments, with 10 mice per group/exp. *** p<0.001, ** p<0.01, * p<0.05, ns p non-significant.

Although antibody titers were decreased by Py infection, they were not eliminated. To determine if compromised antibody response also eliminated MBCs, SRBC-immune mice that were infected or not with Py were subjected to booster immunization with SRBC (Fig 4). Boosting led to a similar fold-increase in SRBC-specific titers and responses occurred with similar kinetics in both groups of mice. These data suggest that MBC responses to SRBC were largely intact in mice that had been infected with Py blood-stage parasites and could be successfully recalled by booster immunization.

### Impact of malaria on pre-existing antibodies to vaccines in children

**Fig 5:**
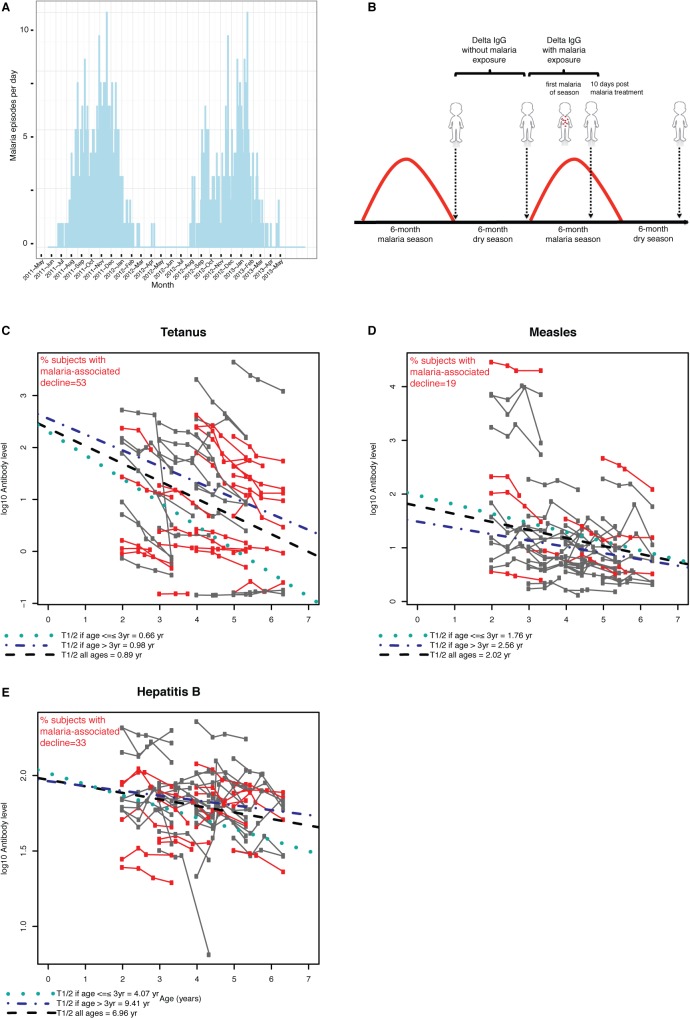
Kinetics of vaccine-specific IgG levels in children during the dry season and after acute malaria. **(A)** The study was designed to take advantage of the sharply demarcated and intense 6-month malaria season (July—December) and 6-month dry season (January—June; negligible malaria transmission) in Mali. Shown is the number of febrile malaria episodes per day over two years at the study site in a cohort of 695 children and adults. **(B)** IgG levels specific for routine vaccines administered under one year of age (tetanus, measles and Hepatitis B) were measured in plasma collected from 54 children at four time points (vertical arrows): before and after the 6-month dry season, 10 days after the first acute malaria episode of the ensuing malaria season, and after the second dry season. Shown for each subject are IgG titers specific for **(C)** tetanus, **(D)** measles and **(E)** hepatitis B vaccines at the time points indicated in **(B)**. The x-axis indicates the age at which the respective time points occurred for each subject. In red are subjects who experienced an accelerated decline in vaccine-specific IgG titers following acute malaria (between 2^nd^ and 3^rd^ time points) relative to each child’s own rate of change during the preceding dry season (between 1^st^ and 2^nd^ time points). The percentage of subjects for whom malaria was associated with an accelerated decline in IgG is shown in red text for each vaccine. A linear mixed effects model that included three time points over 18 months (before and after the first dry season, and after the second dry season) was used to estimate average IgG half-lives for all subjects (black dashed line) and separately for children aged ≤3 years (green dotted line) and >3 years of age (blue dash-dot line).

Together, the observations in mouse models prompted us to investigate the impact of acute malaria on pre-existing antibodies to common vaccines in children. In a longitudinal study in Mali of 54 children aged 2–5 years (mean 3.5 years), we took advantage of the sharply demarcated 6-month rainy season (intense malaria) and 6-month dry season (negligible malaria) [10] (Fig 5A) to compare kinetics of vaccine-specific IgG levels in the presence and absence of malaria exposure. We measured IgG levels to vaccines given to Malian children in the first year of life including tetanus, measles and hepatitis B [17–19]. In Mali, tetanus and hepatitis B vaccines are given at 6, 10 and 14 weeks of age, and the measles vaccine at 9 months [20]. Vaccine-specific IgG was measured at four time points over 18 months: before and after the dry season; 10 days after treatment of the first malaria episode of the ensuing malaria season; and after the subsequent dry season (Fig 5B).

Subjects were asymptomatic and uninfected (by *P*. *falciparum* PCR) at the end of the first dry season. The median time between the end of the first dry season and the first malaria episode was 107 days (IQR 65 days). At the first malaria episode children had an average temperature of 38.4°C (range 37.5–40.3), were infected with *P*. *falciparum* (geometric mean: 33,688 asexual parasites/μl of blood, 95% CI 23,283–47,832) and had no other cause of fever on examination. Malaria was treated with a standard course of artemether/lumefantrine. Demographic/clinical data of subjects are shown in Table 1.

**Table 1:** Demographic and clinical characteristics of study subjects.

Variable	Subjects (n = 54)
Gender, % female (no.)	43 (23)
Age, years [mean (range)]	3.5 (2–5)
Time to first malaria episode, days [median (IQR^1^)] ^2^ ^,^ ^3^	107 (65)
Axillary temperature at first malaria episode, **°**C, [mean (range)]	38.4 (37.5–40.3)
Parasitemia at first malaria, asexual parasites/ μl/blood [geometric mean (95% CI^4^)]	33,688 (23,283–47,832)

^1^Interquartile range.

^2^Malaria episode defined as T≥37.5°C, asexual parasitemia ≥2500/microliter and no other cause of fever discernible on physical examination.

^3^Days since enrollment during a cross-sectional survey before the malaria season in May 2012.

^4^95% confidence interval.

To test the hypothesis that acute malaria accelerates the loss of pre-existing vaccine-specific IgG levels we compared the rate of change in IgG levels during the dry season to the rate of change from the end of the same dry season to 10 days after treatment of the first malaria episode of the ensuing malaria season (Fig 5B). IgG levels ten days post-malaria were measured because mouse models showed heterologous IgG titers fall within 2 weeks of *Plasmodium* infection (Fig 2B). At the population level, the average IgG level slope either remained unchanged (hepatitis B and tetanus) or increased following malaria (measles) relative to average IgG level slopes during the preceding dry season (Table 2); and reassuringly, IgG levels remained above protective thresholds in most children (Table 2). However, at the individual level, malaria was associated with an accelerated decline in vaccine-specific IgG levels in some children, relative to each child’s rate of decline during the preceding dry season (Fig 5C–5E). Specifically, malaria was associated with accelerated declines in IgG specific for tetanus, measles and hepatitis B in 53%, 19% and 33% of children, respectively (Fig 5C–5E). Multivariate analysis revealed a marginally significant relationship between higher temperature at the time of malaria and the risk of accelerated declines in IgG specific for measles (p = 0.07), hepatitis B (p = 0.06) and tetanus (p = 0.07) after adjusting for age, gender, and parasitemia.

**Table 2:** Rate of change of vaccine-specific IgG levels during the dry season versus during malaria exposure and percentage of children with protective IgG levels.

Vac Vaccine	Protective IgG titer	Subjects with protective IgG titer, no. (%)^1^	Dry season slope, log 10 unit/year (SE^2^)	Malaria exposure slope, log10 unit/year (SE)	p value^3^
Tetanus	0.01 IU/mL [21]	54 (100)	-0.247 (0.054)^4^	-0.394 (0.091) ^4^	0.48
Measles	0.2 IU/mL [22]	54 (100)	-0.125 (0.052) ^4^	0.288 (0.087) ^4^	0.003
Hepatitis B	10 mIU/mL [23]	52 (96)	-0.037 (0.023)	0.229 (0.122)	0.35

^1^Results based on the last time point of the study period.

^2^Standard error.

^3^p value for the difference in slopes between the dry season and period of malaria exposure was obtained by fitting a linear mixed effects model.

^4^Indicates that the slope is significantly different than zero.

In light of these findings, and given that children in this region experience up to 5 malaria episodes/season [24], we performed exploratory analyses to estimate vaccine-specific IgG half-lives using data from three time points: before and after the first dry season, and after the second dry season (Fig 5B). The mean half-life of IgG levels for tetanus was 0.89 years (95% CI, 0.70–1.21), measles 2.02 years (95% CI, 1.57–2.83) and hepatitis B 6.96 years (95% CI, 4.60–14.33) (Table 3; Fig 5C–5E). Since previous studies showed that antibody responses do not reach steady-state levels until 3–4 years after vaccination [8, 25, 26] we estimated IgG half-lives separately for children aged 2–3 years or 4–5 years at study entry, the latter providing a closer approximation of steady-state levels (~3–4 years post-vaccination). As expected, IgG half-life estimates were greater in older children—tetanus 0.98 years (95% CI, 0.78–1.34), measles 2.56 years (95% CI, 1.74–4.91) and hepatitis B 9.41 years (95% CI, 5.15–54.31) (Table 3; Fig 5C–5E).

**Table 3:** Half-life estimates of vaccine-specific IgG levels overall and by age group.

VaccineV Vaccine	All		Age ≤ 3 years		Age > 3 years	
	Slope^1^ (SE^2^)	T _½_ ^3^,years	Slope (SE)	T _½_, years	Slope (SE)	T _½_, years
Tetanus	-0.34 (0.05)	0.89 (0.70, 1.21)	-0.45 (0.10)	0.66 (0.47, 1.14)	-0.31 (0.04)	0.98 (0.78, 1.34)
Measles	-0.15 (0.02)	2.02 (1.57, 2.83)	-0.17 (0.03)	1.76 (1.26, 2.90)	-0.12 (0.03)	2.56 (1.74, 4.91)
Hepatitis B	-0.04 (0.01)	6.96 (4.60, 14.33)	-0.07 (0.02)	4.07 (2.62, 9.17)	-0.03 (0.01)	9.41 (5.15, 54.31)

^1^Slopes are the change in mean log10 antibody titer per year estimated in the linear mixed effects model with random intercept.

^2^Standard error.

^3^Half-life.

## Discussion

Here we provide evidence in mice and some children that *Plasmodium* infection is associated with an accelerated loss of pre-existing viral and vaccine-specific antibodies. We extend the recent study of Ng et al. with the *Pcc* model [15] and describe marked and sustained decreases in antibody levels in *Py*-infected inbred and outbred mice. Reassuringly, population-averaged IgG levels to tetanus, measles and hepatitis B in children did not decrease with acute malaria and remained above protective thresholds in most children during the study period. However, population-averaged data obscures the observation that acute malaria was associated with accelerated losses of vaccine-specific IgG in some children, particularly for tetanus, which may have public health implications. Indeed, a recent report from Mali (<60km from study site) indicated that 6.5% of hospital admissions were for tetanus with a mortality rate of 46.2% [27]. Moreover, exploratory analyses yielded half-life estimates of IgG specific for tetanus (0.98 years) and measles (2.56 years) that were considerably shorter than estimates reported in a study of U.S. adults (tetanus: 11 years; measles: 3014 years) [8], further suggesting that chronic malaria exposure and associated factors (co-infection, malnutrition) may accelerate the loss of vaccine-specific antibodies. As expected, we found that vaccine-specific IgG half-life estimates were greater in older children who were immunized 3–4 years before this study—a point at which antibody decay rates approach steady-state [25, 26]. However, it remains possible that the steady-state had not been reached in these children and additional studies are needed to resolve this.

Mice used in this study were malaria-naïve, whereas children had already experienced 2–5 malaria seasons; therefore, it remains possible that *P*. *falciparum* infections earlier in life would accelerate the loss of vaccine-specific IgG in more children and to a greater degree than observed here. Controlled human malaria infections [28] could determine the impact of first *P*. *falciparum* infections on pre-existing levels of vaccine-specific antibodies.

Amanna et al. found that smallpox vaccination (vaccinia virus infection) in humans did not significantly alter levels of pre-existing IgG to unrelated antigens, including tetanus and measles [29], suggesting that the accelerated declines in vaccine-specific IgG observed in association with acute malaria in this study may be specific to *Plasmodium* rather than a general feature of infection. Accordingly, we did not detect a decline in IgG to unrelated antigens in mice after infection with *Listeria monocytogenes* or LCMV (Fig 1B).

The mechanisms by which *Plasmodium* accelerates the decline of IgG to unrelated antigens remains unclear. It has been suggested that binding of *Plasmodium*-infected erythrocytes to bone marrow stromal cells may disrupt LLPC survival signals [30, 31]. Additionally, we find that BAFF receptor expression is downmodulated on splenic and bone marrow plasma cells and circulating BAFF levels increase in *Py*-infected mice (Fig 3), the latter is a phenomenon also observed in human malaria [32]. The recent study with *Pcc* suggested that *Plasmodium*-induced polyclonal B cell activation and the attending hypergammaglobulinemia resulted in apoptosis of LLPC through a CD32-dependent mechanism (21). Consistent with this, we observed substantial increases in apoptotic plasma cells in the bone marrow of mice after *Py* infection (Fig 3). Thus, the decline in LLPCs observed in mice after *Plasmodium* infection may be multifactorial.

We observed that acute malaria is associated with a transient increase in vaccine-specific IgG in some children. It is possible that bystander proliferation and differentiation of vaccine-specific MBCs into ASCs [33] masked the loss of vaccine-specific LLPCs in some children. This is supported by prior observations that acute malaria is associated with modest increases in tetanus-specific MBCs [34], and by *in vitro* studies that implicate the cysteine-rich interdomain regions 1α of the *P*. *falciparum* erythrocyte membrane protein 1 (PfEMP1) as a T cell–independent polyclonal B cell activator [35]. The factors underlying net increases or decreases in antibody levels to unrelated antigens during acute malaria in children are unclear. We found a marginally significant association between higher temperature during malaria and declines in IgG to tetanus, measles and hepatitis B; whereas age, gender and parasitemia had no effect. Additional studies may illuminate other factors underlying the variable effects of malaria on pre-existing heterologous antibodies.

Approximately 3.4 billion people in 97 countries are at risk of malaria [36]. Here we provide evidence that acute malaria is associated with an accelerated loss of pre-existing viral and vaccine-specific IgG in mice and some children. Given the enormous burden of malaria worldwide, even modest malaria-associated declines in vaccine-specific antibodies in a fraction of the population could contribute to the high incidence of vaccine-preventable diseases. Further studies are needed to investigate the generalizability of these findings and their potential implications for vaccine policies in malaria-endemic countries.

## References

[1] Keja K, Chan C, Hayden G, Henderson RH. Expanded programme on immunization. World health statistics quarterly Rapport trimestriel de statistiques sanitaires mondiales. 1988;41(2):59–63. PubMed . 3176515

[2] Okwo-Bele JM, Cherian T. The expanded programme on immunization: a lasting legacy of smallpox eradication. Vaccine. 2011;29 Suppl 4:D74–9. 10.1016/j.vaccine.2012.01.080 PubMed . 22486980

[3] Black RE, Cousens S, Johnson HL, Lawn JE, Rudan I, Bassani DG, et al Global, regional, and national causes of child mortality in 2008: a systematic analysis. Lancet. 2010;375(9730):1969–87. 10.1016/S0140-6736(10)60549-1 PubMed . 20466419

[4] Centers for Disease C, Prevention. Global routine vaccination coverage, 2011. MMWR Morbidity and mortality weekly report. 2012;61(43):883–5. PubMed . 23114256

[5] Greenwood BM, Bradley-Moore AM, Bryceson AD, Palit A. Immunosuppression in children with malaria. Lancet. 1972;1(7743):169–72. PubMed . 4109547 10.1016/s0140-6736(72)90569-7

[6] Plotkin SA. Correlates of protection induced by vaccination. Clin Vaccine Immunol. 2010;17(7):1055–65. 10.1128/CVI.00131-10 PubMed 20463105 PMC2897268

[7] Tarlinton D, Good-Jacobson K. Diversity among memory B cells: origin, consequences, and utility. Science. 2013;341(6151):1205–11. 10.1126/science.1241146 PubMed . 24031013

[8] Amanna IJ, Carlson NE, Slifka MK. Duration of humoral immunity to common viral and vaccine antigens. N Engl J Med. 2007;357(19):1903–15. PubMed . 17989383 10.1056/NEJMoa066092

[9] Crompton PD, Kayala MA, Traore B, Kayentao K, Ongoiba A, Weiss GE, et al A prospective analysis of the Ab response to Plasmodium falciparum before and after a malaria season by protein microarray. Proc Natl Acad Sci U S A. 2010;107(15):6958–63. 10.1073/pnas.1001323107 PubMed 20351286 PMC2872454

[10] Tran TM, Li S, Doumbo S, Doumtabe D, Huang CY, Dia S, et al An intensive longitudinal cohort study of Malian children and adults reveals no evidence of acquired immunity to Plasmodium falciparum infection. Clin Infect Dis. 2013;57(1):40–7. 10.1093/cid/cit174 PubMed 23487390 PMC3669526

[11] Portugal S, Pierce SK, Crompton PD. Young lives lost as B cells falter: what we are learning about antibody responses in malaria. J Immunol. 2013;190(7):3039–46. 10.4049/jimmunol.1203067 PubMed . 23526829 PMC3608210

[12] Butler NS, Moebius J, Pewe LL, Traore B, Doumbo OK, Tygrett LT, et al Therapeutic blockade of PD-L1 and LAG-3 rapidly clears established blood-stage Plasmodium infection. Nat Immunol. 2011;13(2):188–95. Epub 2011/12/14. ni.2180 [pii] 10.1038/ni.2180 PubMed . 22157630 PMC3262959

[13] Weiss GE, Crompton PD, Li S, Walsh LA, Moir S, Traore B, et al Atypical memory B cells are greatly expanded in individuals living in a malaria-endemic area. J Immunol. 2009;183(3):2176–82. Epub 2009/07/14. jimmunol.0901297 [pii] 10.4049/jimmunol.0901297 PubMed . 19592645 PMC2713793

[14] Illingworth J, Butler NS, Roetynck S, Mwacharo J, Pierce SK, Bejon P, et al Chronic exposure to Plasmodium falciparum is associated with phenotypic evidence of B and T cell exhaustion. J Immunol. 2013;190(3):1038–47. 10.4049/jimmunol.1202438 PubMed 23264654 PMC3549224

[15] Ng DH, Skehel JJ, Kassiotis G, Langhorne J. Recovery of an antiviral antibody response following attrition caused by unrelated infection. PLoS Pathog. 2014;10(1):e1003843 10.1371/journal.ppat.1003843 PubMed 24391499 PMC3879355

[16] Rickert RC, Jellusova J, Miletic AV. Signaling by the tumor necrosis factor receptor superfamily in B-cell biology and disease. Immunol Rev. 2011;244(1):115–33. 10.1111/j.1600-065X.2011.01067.x PubMed 22017435 PMC3202302

[17] Measles vaccines: WHO position paper. Releve epidemiologique hebdomadaire / Section d'hygiene du Secretariat de la Societe des Nations = Weekly epidemiological record / Health Section of the Secretariat of the League of Nations. 2009;84(35):349–60. PubMed . 19714924

[18] Hepatitis B vaccines. Releve epidemiologique hebdomadaire / Section d'hygiene du Secretariat de la Societe des Nations = Weekly epidemiological record / Health Section of the Secretariat of the League of Nations. 2009;84(40):405–19. PubMed . 19817017

[19] Tetanus vaccine. Releve epidemiologique hebdomadaire / Section d'hygiene du Secretariat de la Societe des Nations = Weekly epidemiological record / Health Section of the Secretariat of the League of Nations. 2006;81(20):198–208. PubMed . 16710950

[20] WHO. Immunization Summary. 2012:106.

[21] Wolters KL, Dehmel H. Abschlies- sende untersuchungen uber die Tetanus Prophylaxe durch active Immunisierung. Zeitschrift fur Hyeitschrift. 1942;124:326–32.

[22] Chen RT, Markowitz LE, Albrecht P, Stewart JA, Mofenson LM, Preblud SR, et al Measles antibody: reevaluation of protective titers. J Infect Dis. 1990;162(5):1036–42. PubMed . 2230231 10.1093/infdis/162.5.1036

[23] Jack AD, Hall AJ, Maine N, Mendy M, Whittle HC. What level of hepatitis B antibody is protective? J Infect Dis. 1999;179(2):489–92. 10.1086/314578 PubMed . 9878036

[24] Crompton PD, Traore B, Kayentao K, Doumbo S, Ongoiba A, Diakite SA, et al Sickle Cell Trait Is Associated with a Delayed Onset of Malaria: Implications for Time-to-Event Analysis in Clinical Studies of Malaria. J Infect Dis. 2008;198(9):1265–75. Epub 2008/08/30. 10.1086/592224 PubMed . 18752444 PMC2574881

[25] Dai B, Chen ZH, Liu QC, Wu T, Guo CY, Wang XZ, et al Duration of immunity following immunization with live measles vaccine: 15 years of observation in Zhejiang Province, China. Bull World Health Organ. 1991;69(4):415–23. PubMed 1934235 PMC2393239

[26] Amanna IJ, Slifka MK. Mechanisms that determine plasma cell lifespan and the duration of humoral immunity. Immunol Rev. 2010;236:125–38. 10.1111/j.1600-065X.2010.00912.x PubMed . 20636813 PMC7165522

[27] Minta DK, Traore AM, Soucko AK, Dembele M, Coulibaly Y, Dicko MS, et al [Mortality and morbidity of tetanus in the infectious diseases department, Point G teaching hospital, in Bamako, Mali (2004–2009)]. Bulletin de la Societe de pathologie exotique. 2012;105(1):58–63. 10.1007/s13149-011-0204-y PubMed . 22228429

[28] Sauerwein RW, Roestenberg M, Moorthy VS. Experimental human challenge infections can accelerate clinical malaria vaccine development. Nat Rev Immunol. 2011;11(1):57–64. Epub 2010/12/24. nri2902 [pii] 10.1038/nri2902 PubMed . 21179119

[29] Amanna IJ, Hammarlund E, Lewis MW, Slifka MK. Impact of infection or vaccination on pre-existing serological memory. Human immunology. 2012;73(11):1082–6. 10.1016/j.humimm.2012.07.328 PubMed 22902392 PMC3478407

[30] Kinyanjui SM, Conway DJ, Lanar DE, Marsh K. IgG antibody responses to Plasmodium falciparum merozoite antigens in Kenyan children have a short half-life. Malar J. 2007;6:82 Epub 2007/06/30. 1475-2875-6-82 [pii] 10.1186/1475-2875-6-82 PubMed . 17598897 PMC1920526

[31] Rogers NJ, Hall BS, Obiero J, Targett GA, Sutherland CJ. A model for sequestration of the transmission stages of Plasmodium falciparum: adhesion of gametocyte-infected erythrocytes to human bone marrow cells. Infect Immun. 2000;68(6):3455–62. Epub 2000/05/19. PubMed . 10816498 10.1128/iai.68.6.3455-3462.2000 PMC97624

[32] Nduati E, Gwela A, Karanja H, Mugyenyi C, Langhorne J, Marsh K, et al The plasma concentration of the B cell activating factor is increased in children with acute malaria. J Infect Dis. 2011;204(6):962–70. Epub 2011/08/19. jir438 [pii] 10.1093/infdis/jir438 PubMed . 21849293 PMC3156925

[33] Bernasconi NL, Traggiai E, Lanzavecchia A. Maintenance of serological memory by polyclonal activation of human memory B cells. Science. 2002;298(5601):2199–202. 10.1126/science.1076071 PubMed . 12481138

[34] Weiss GE, Traore B, Kayentao K, Ongoiba A, Doumbo S, Doumtabe D, et al The Plasmodium falciparum-specific human memory B cell compartment expands gradually with repeated malaria infections. PLoS Pathog. 2010;6(5):e1000912 Epub 2010/05/27. 10.1371/journal.ppat.1000912 PubMed . 20502681 PMC2873912

[35] Donati D, Mok B, Chene A, Xu H, Thangarajh M, Glas R, et al Increased B cell survival and preferential activation of the memory compartment by a malaria polyclonal B cell activator. J Immunol. 2006;177(5):3035–44. Epub 2006/08/22. 177/5/3035 [pii]. PubMed . 16920940 10.4049/jimmunol.177.5.3035

[36] WHO. World Malaria Report 2013. 2013:284.

